# Supplementary material for: Analysis of natural structures and chemical mapping data reveals local stability compensation in RNA
Source: bioRxiv. 2025 Apr 11:2024.12.11.627843. Originally published 2024 Dec 12. Preprint. [Version 2] doi: 10.1101/2024.12.11.627843 (PMC11661157; doi:10.1101/2024.12.11.627843)
Supplement: Supplement 1 [file media-1.pdf]

Supplementary Information

Supplementary Figure 1..1
Supplementary Figure 2..2
Supplementary Table 1..3
Supplementary Table 2..4
Supplementary Figure 3..5
Supplementary Table 3..6
Supplementary Figure 4..7
Supplementary Figure 5..8
Supplementary Table 4..9
Supplementary Figure 6..11
Supplementary Figure 7..12
Supplementary Table 5..13

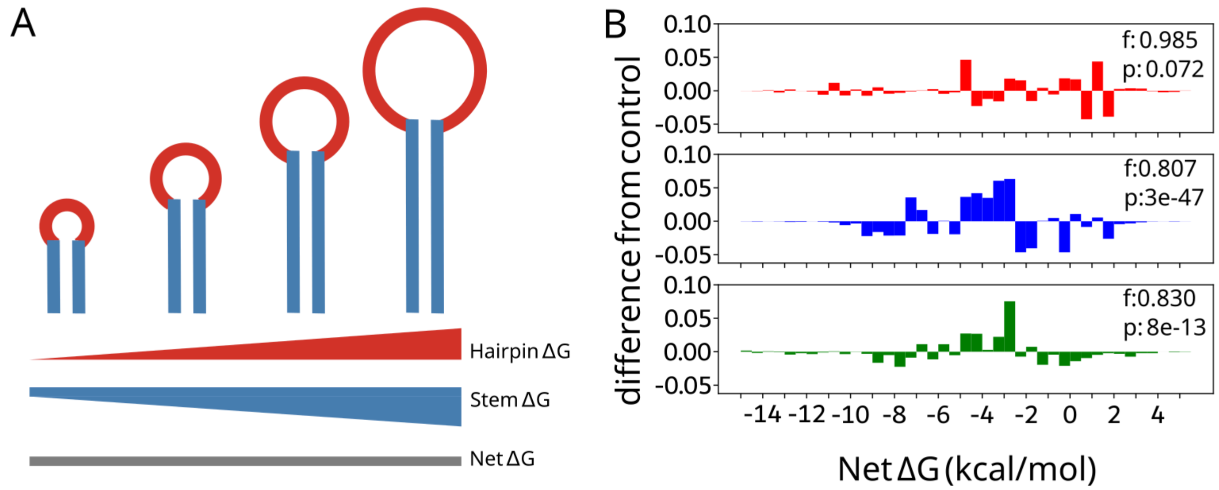

Supplementary Figure 1. Local stability compensation against an in-structure rotation control.(A), Schematic demonstrating local stability compensation, which proposes that larger hairpins are closed by larger stems to maintain the net free energy. (B) Diagram explaining stem swapping in the rotation control (C) The difference in net ΔG distribution between bpRNA-1m90 refolded structures versus rotated structures for hairpins (red)(p=0.072), bulges (blue)(p=3e-47), and internal loops (green)(p=8e-13).

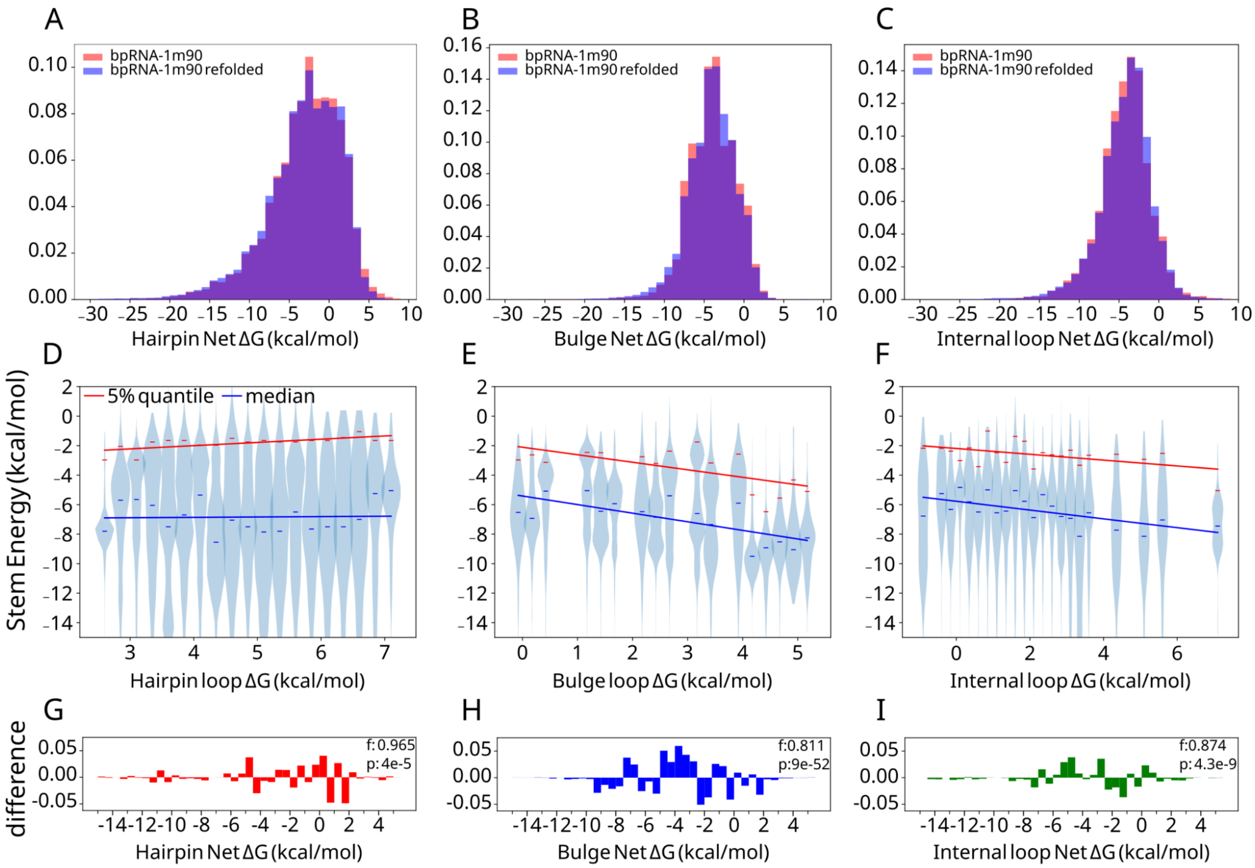

Supplementary Figure 2. Comparing refolded data with bpRNA-1m90. (A) Hairpin (B) bulge and (C) internal loop net ΔG densities for bpRNA-1m90 and bpRNA-1m90 including refolded structures. (D) Hairpin, (E) bulge, and (F) internal loop ΔGs are binned and the corresponding stem ΔGs are plotted as violins with linear regressions drawn for the median (blue) and 5% quantile (red). (G) The difference in net ΔG distribution between bpRNA-1m90 structures versus rotated structures for hairpins (red)(p=4e-5), (H) bulges (blue)(p=9e-52), and (I) internal loops (green)(p=4.3e-9).

	Hairpins	Bulges	Internal Loops
5% quantile refolded	0.370	0.412	0.456
median refolded	0.401	0.647	0.631
5% quantile	0.451	0.418	0.245
median	0.001	0.506	0.404

Supplementary Table 1. Correlations of stem ΔG median and 5% quantile in refolded data and bpRNA-1m90.

	Hairpins	Bulges	Internal Loops
Mean plotted RNA classes	-2.66	-4.29	-3.85
Stdev plotted RNA classes	1.74	0.75	1.33
Mean all subclasses	-2.64	-4.90	-4.59
Stdev all subclasses	5.44	3.35	3.77

Supplementary Table 2. Average and standard deviation of RNA category medians. Plotted RNA classes include only the major RNA classes included in figure 1.

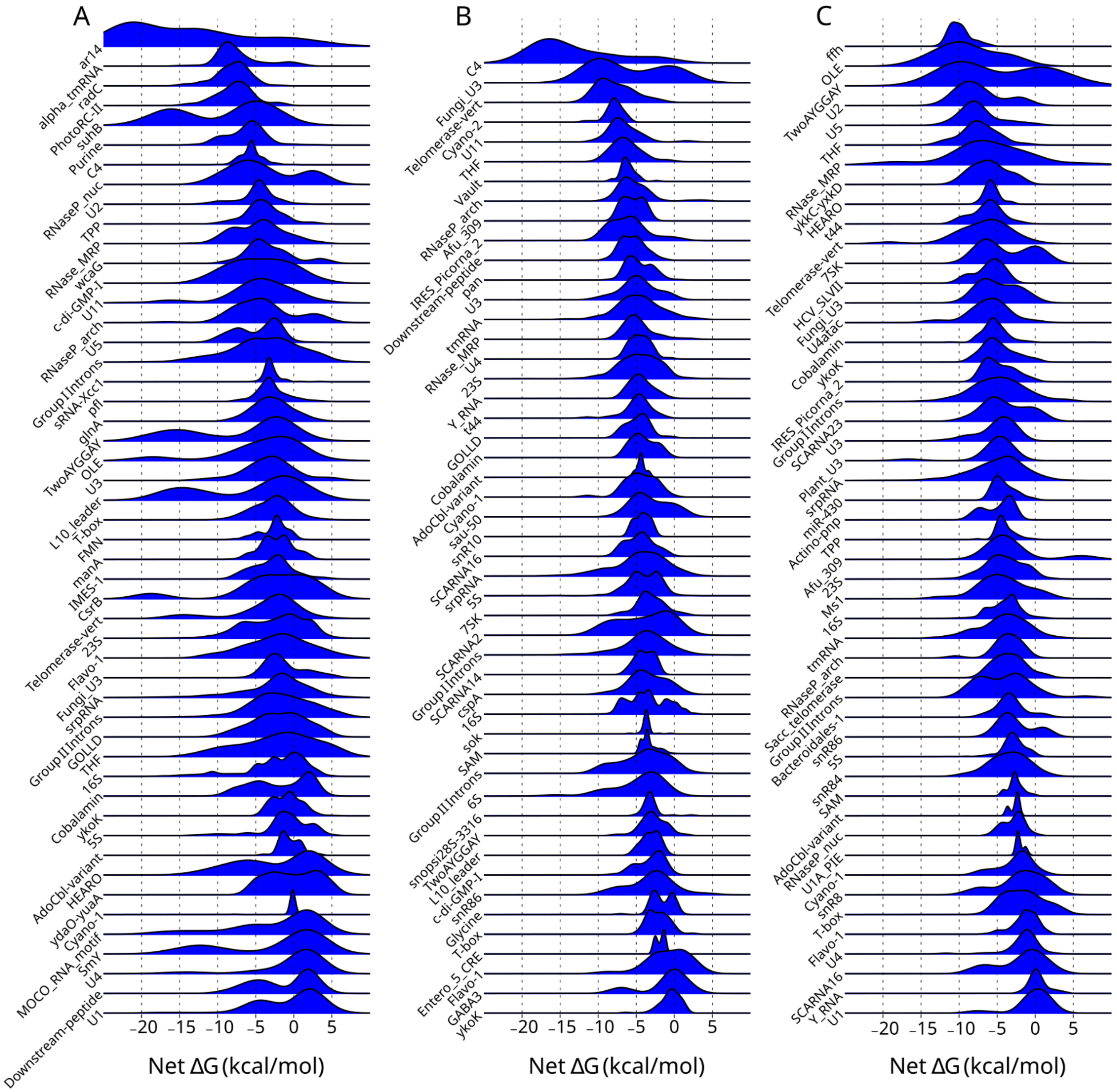

Supplementary Figure 3. Net ΔG variation by RNA subtype in bpRNA-1m90. Subtypes sorted by median net ΔG and the corresponding net ΔG distributions are plotted for (A) hairpins, (B) bulges, and (C) internal loops.

	Hairpins (pre-refold)	Hairpins	Bulges	Internal loops
KS statistic	0.207	0.166	0.088	0.195
p value	0.000	0.000	0.035	0.000

Supplementary Table 3. plant pre-miRNA net ΔGs are distinct from those of other species.

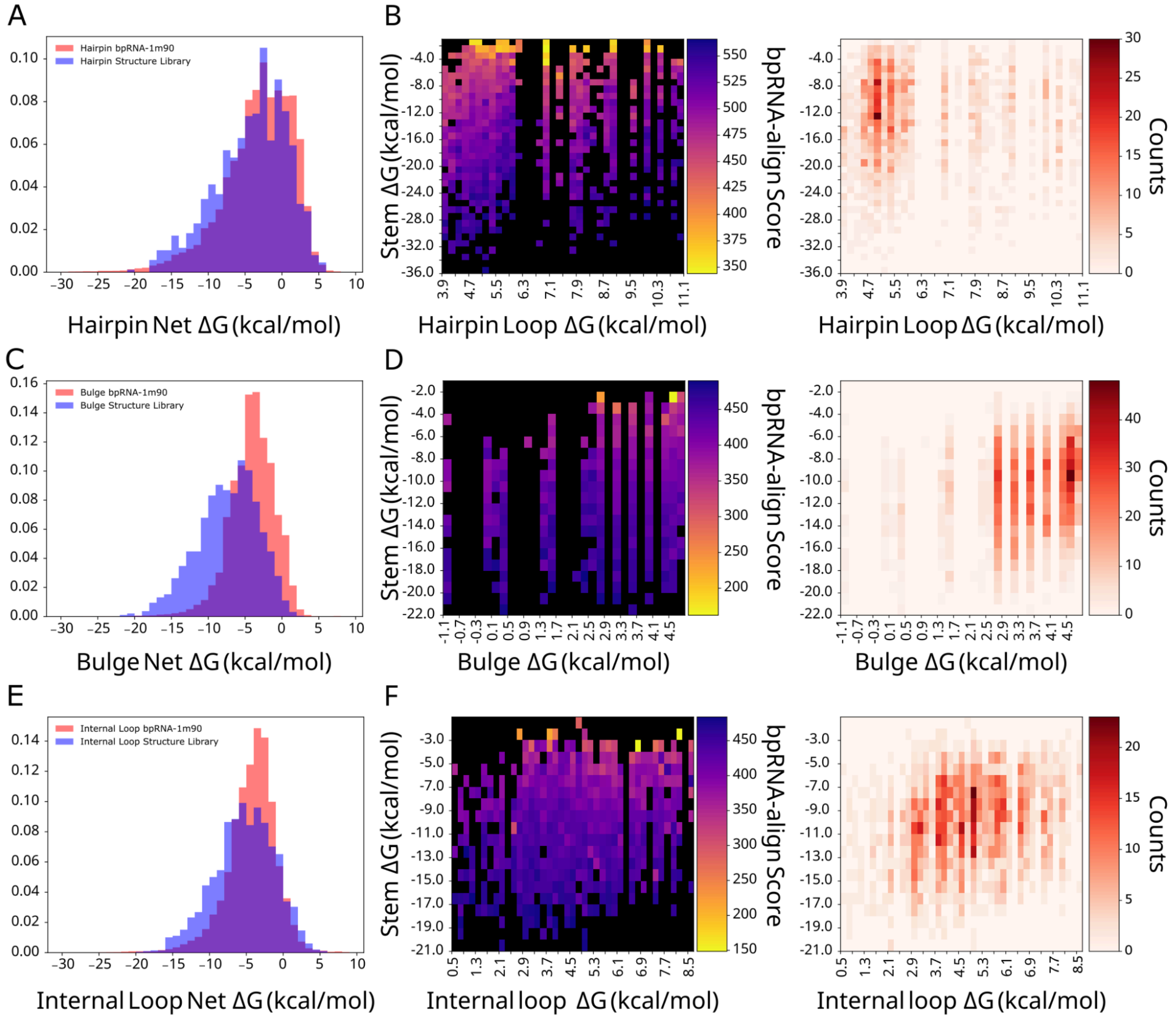

Supplementary Figure 4. Library validation and RNAfold structural similarity heatmaps. (A) Hairpin library net ΔG overlap with refolded RNAs from bpRNA-1m90, (B) Structure alignment score heatmaps for library structure hairpin and stem ΔG, sample sizes for each cell. (C) Bulge library net ΔG overlap with bpRNA-1m90, (D) Structure alignment score heatmaps for library structure bulge and stem ΔG, sample sizes for each cell. (E) Internal loop library net ΔG overlap with bpRNA-1m90, (F) Structure alignment score heatmaps for library structure internal loop and stem ΔG, sample sizes for each cell. Heatmap cells colored yellow have poor alignment while cells colored blue have good alignment.

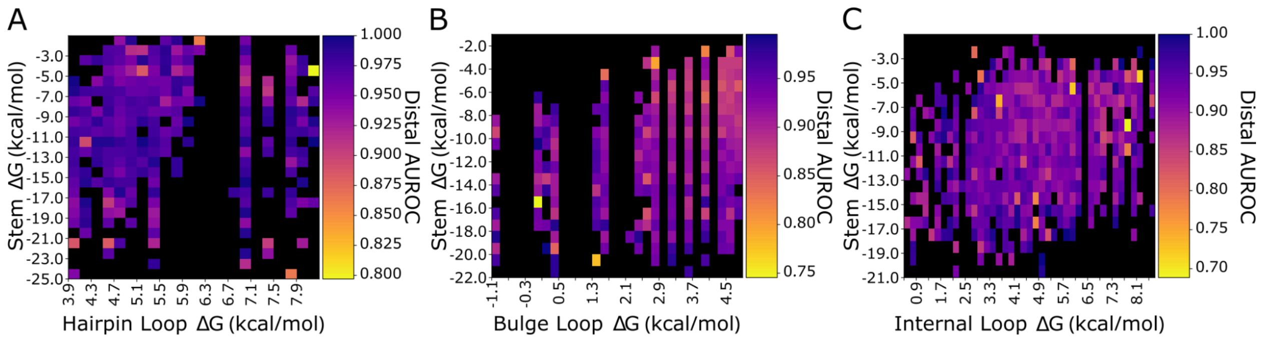

Supplementary Figure 5. Non-local AUROC is not modulated by local stem or loop ΔG. AUROC heatmaps for the distal structures of (A) Hairpin loops, (B) bulges, and (C) internal loops.

A) Local AUROC	Loop ΔG R^2	p	Stem ΔG R^2	p	Net ΔG R^2	p
Hairpins	0.055	3e-12	0.226	3e-50	0.260	7e-59
Bulges	0.068	5e-32	0.187	5e-91	0.211	1e-103
Internal Loops	0.069	1e-32	0.179	7e-87	0.206	1e-101

B) Local stem reactivity	Loop ΔG R^2	p	Stem ΔG R^2	p	Net ΔG R^2	p
Hairpins	0.045	3e-10	0.426	7e-107	0.458	9e-118
Bulges	0.104	3e-49	0.219	4e-108	0.258	4e-130
Internal Loops	0.083	3e-39	0.295	7e-153	0.318	2e-167

Supplementary Table 4. Net ΔG is best correlated with folding metrics. (A) Local AUROC correlation coefficients corresponding to loop, stem, and net free energy. (B) Local stem reactivity correlation coefficients corresponding to loop, stem, and net free energy.

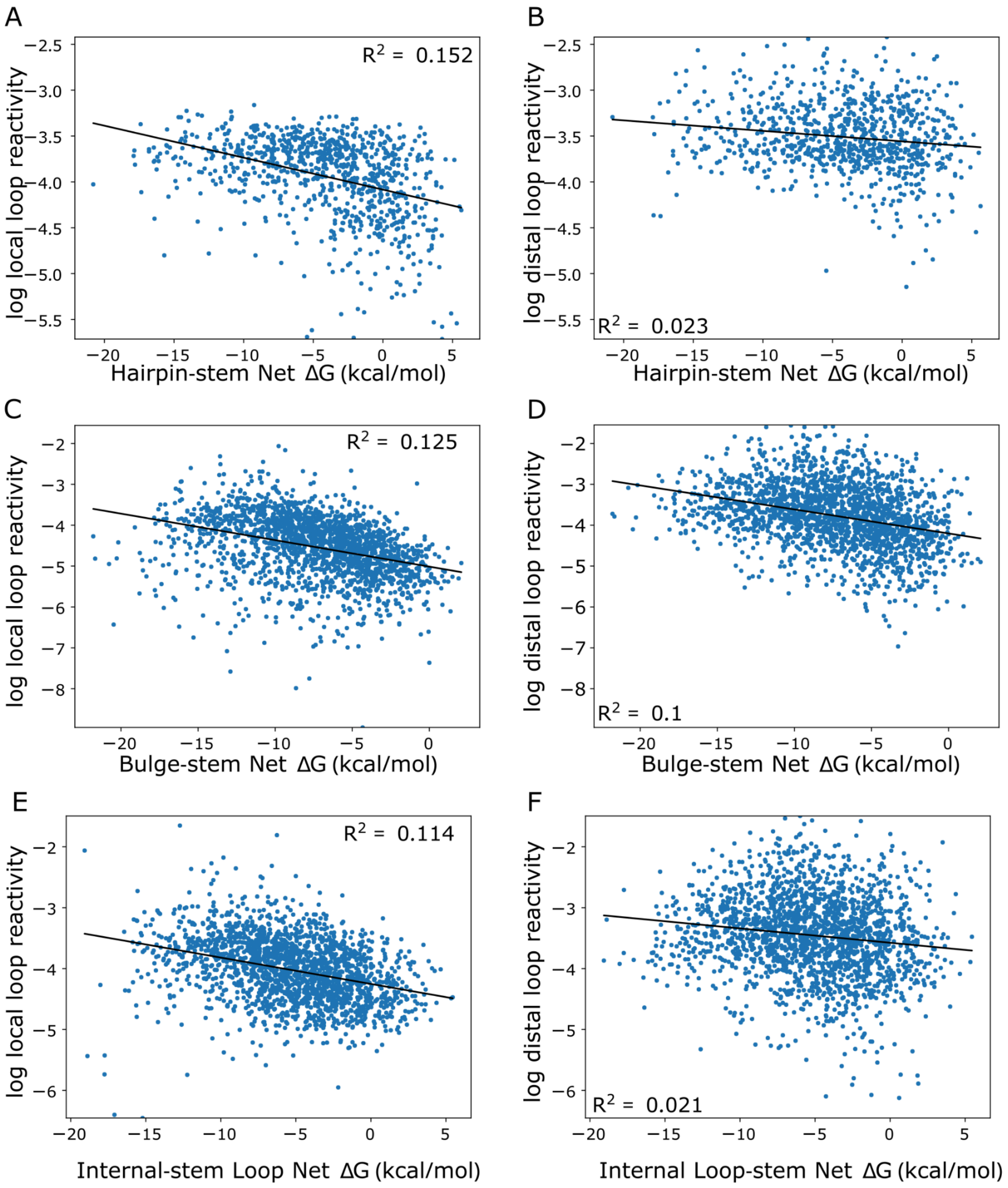

Supplementary Figure 6. Loop reactivities are invariant to loop net ΔGs. Hairpin-stem net ΔG versus local (A) and distal (B) average loop reactivities. Bulge-stem net ΔG versus local (C) and distal (D) average loop reactivities. Internal loop-stem net ΔG versus local (E) and distal (F) average loop reactivities.

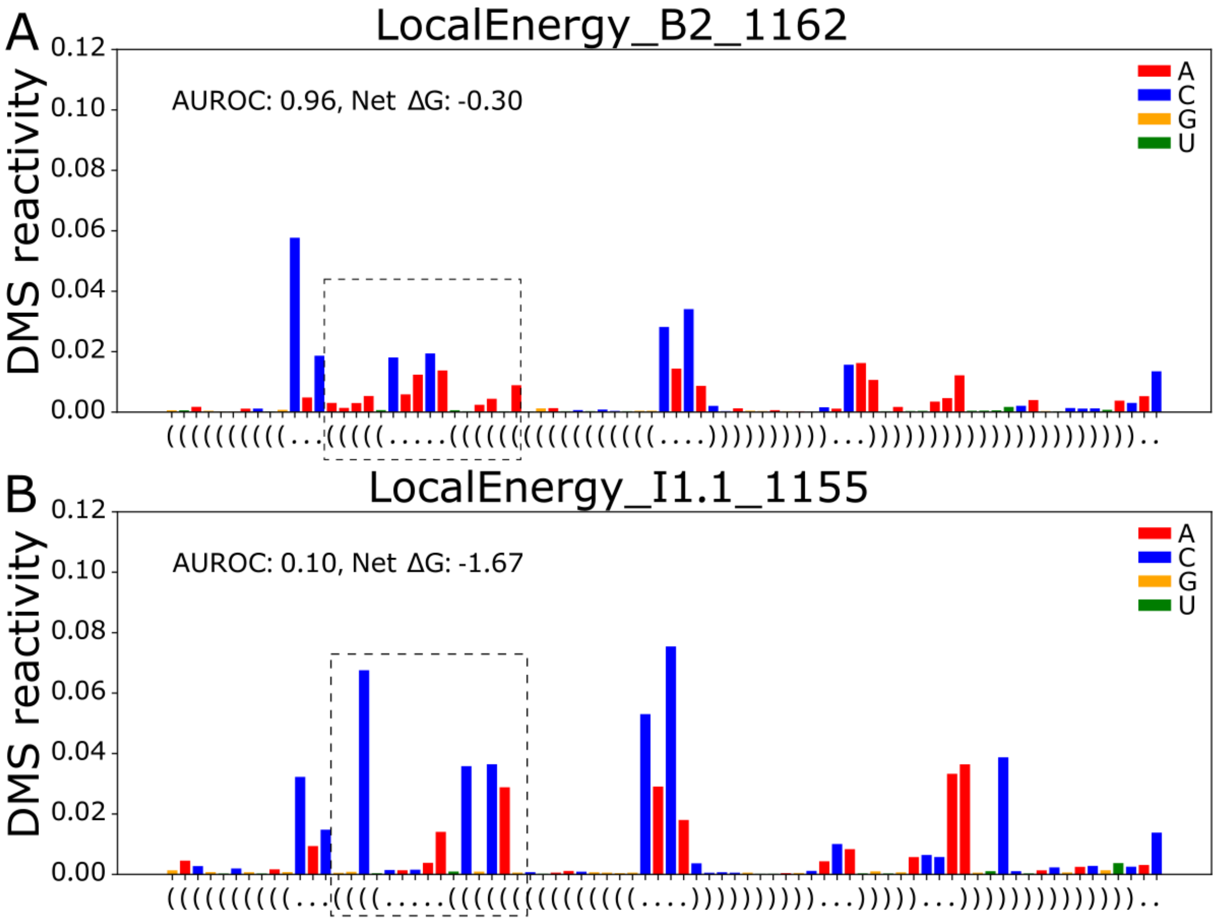

Supplementary Figure 7. Designed structure reactivity examples. (A) example structure from the bulge library with the bulge and adjacent stems highlighted. (B) an example internal loop showing loop shifting in the highlighted region.

	K	n	A
Hairpin loops	18.6	4.10	1.
Bulges	17.4	2.58	1.
Internal loops	20.7	2.95	1.

Supplementary Table 5. Fit parameters for curve fitting to the Hill Equation.
