# Supplementary material for: Analysis of natural structures and chemical mapping data reveals local stability compensation in RNA
Source: bioRxiv. 2024 Dec 12:2024.12.11.627843. Preprint. [Version 1] doi: 10.1101/2024.12.11.627843 (PMC11661157; doi:10.1101/2024.12.11.627843)
Supplement: Supplementary file 1 [file media-2.pdf]

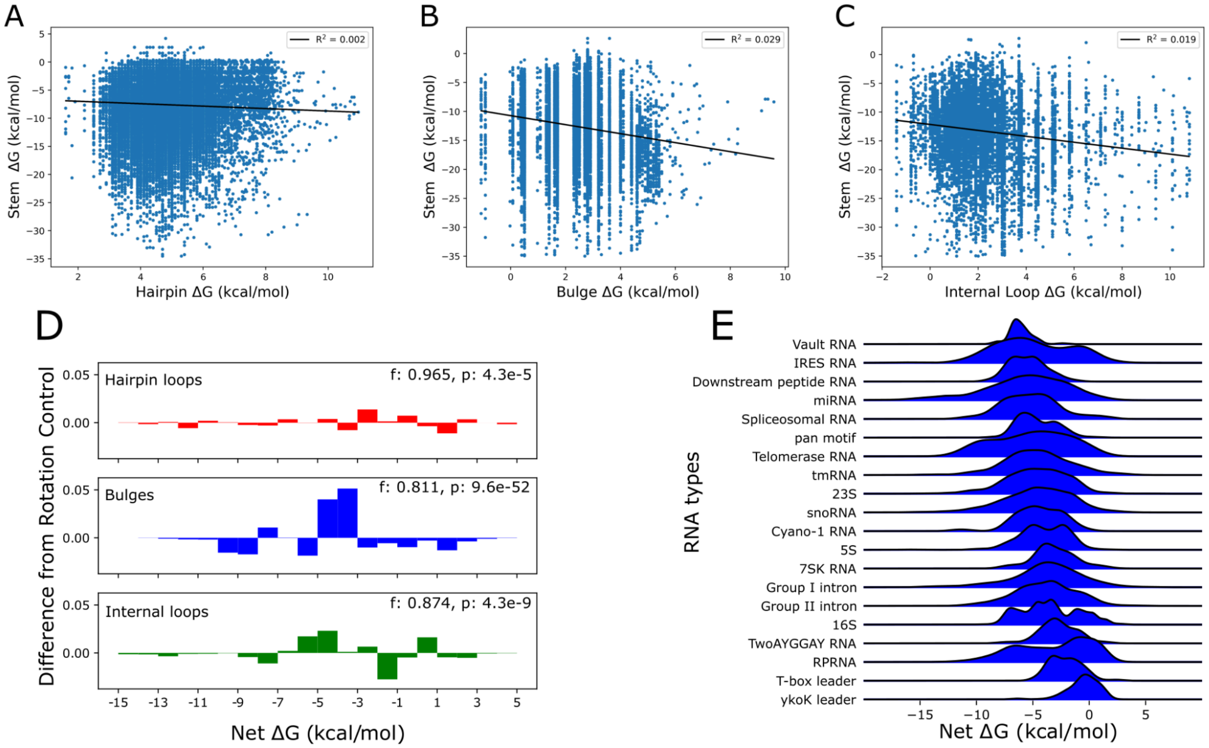

Supplementary Figure 1. Differences in variation globally and by RNA type. bpRNA-1m90 hairpin loop (A), bulge (B), and internal loop (C) ΔGs versus stem ΔGs. (D) Frequency differences between bpRNA-1m90 and a stem/loop rotation control (see materials and methods). (E) Net ΔG distributions for the most abundant bulges in bpRNA-1m90.

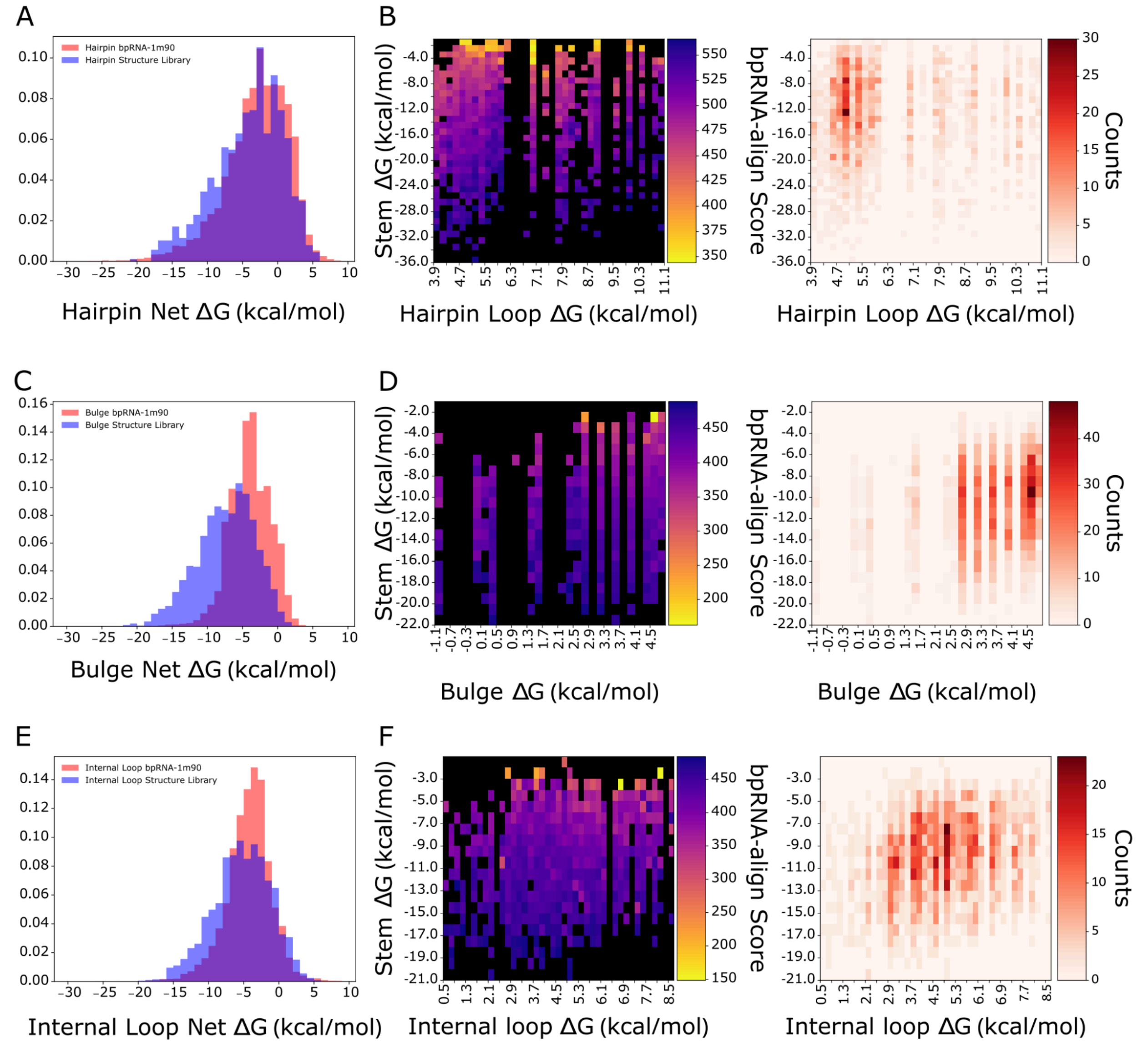

Supplementary Figure 2. Library validation and RNAfold structural similarity heatmaps. (A) Hairpin library net ΔG overlap with bpRNA-1m90, (B) Structure alignment score heatmaps for library structure hairpin and stem ΔG, sample sizes for each cell. (C) Bulge library net ΔG overlap with bpRNA-1m90, (D) Structure alignment score heatmaps for library structure bulge and stem ΔG, sample sizes for each cell. (E) Internal loop library net ΔG overlap with bpRNA-1m90, (F) Structure alignment score heatmaps for library structure internal loop and stem ΔG, sample sizes for each cell.

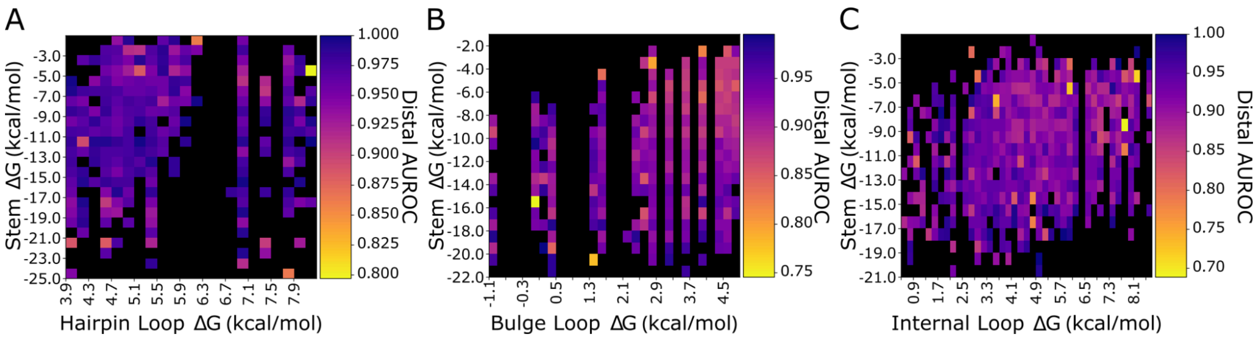

Supplementary Figure 3. Non-local AUROC is not modulated by local stem or loop ΔG. AUROC heatmaps for the distal structures of (A) Hairpin loops, (B) bulges, and (C) internal loops.

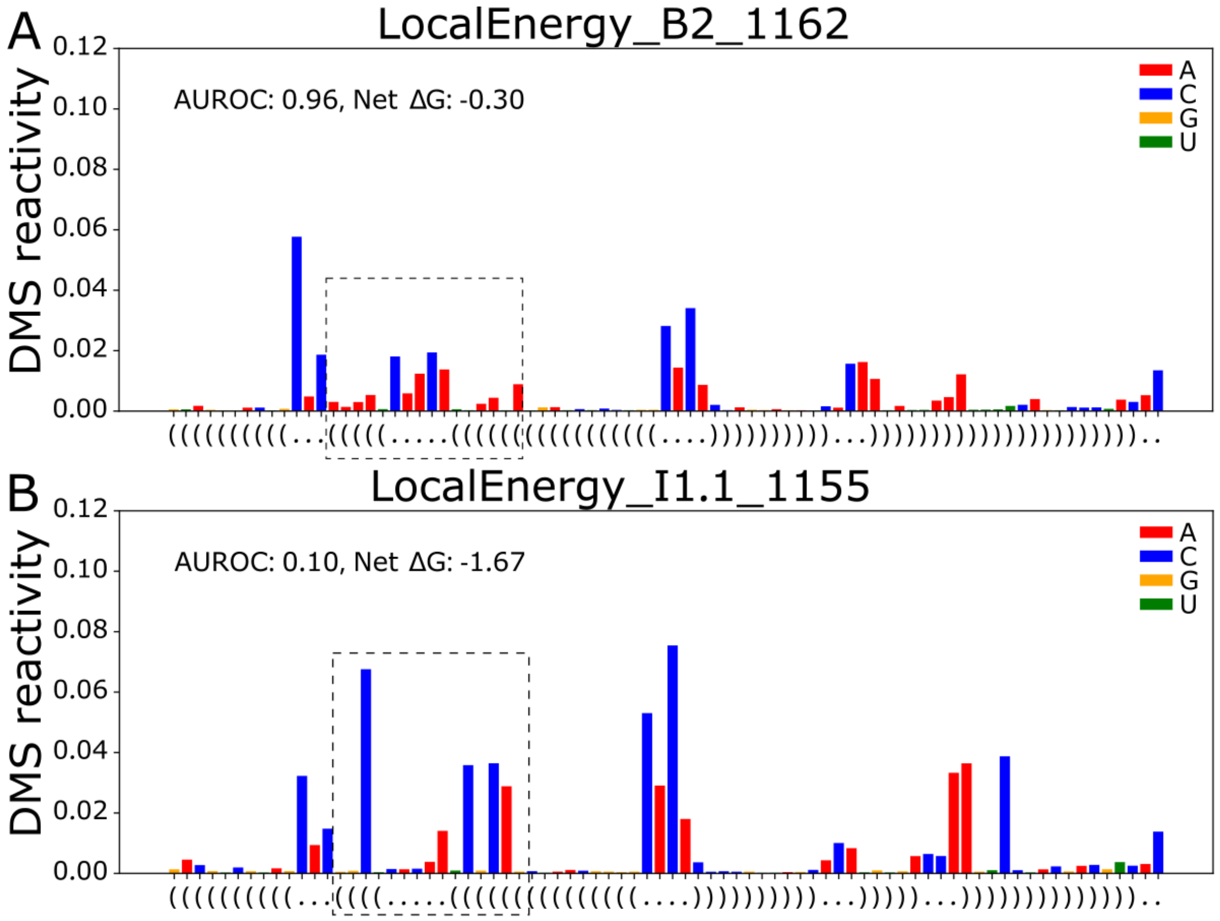

Supplementary Figure 4. Designed structure reactivity examples. (A) example structure from the bulge library with the bulge and adjacent stems highlighted. (B) an example internal loop showing loop shifting in the highlighted region.

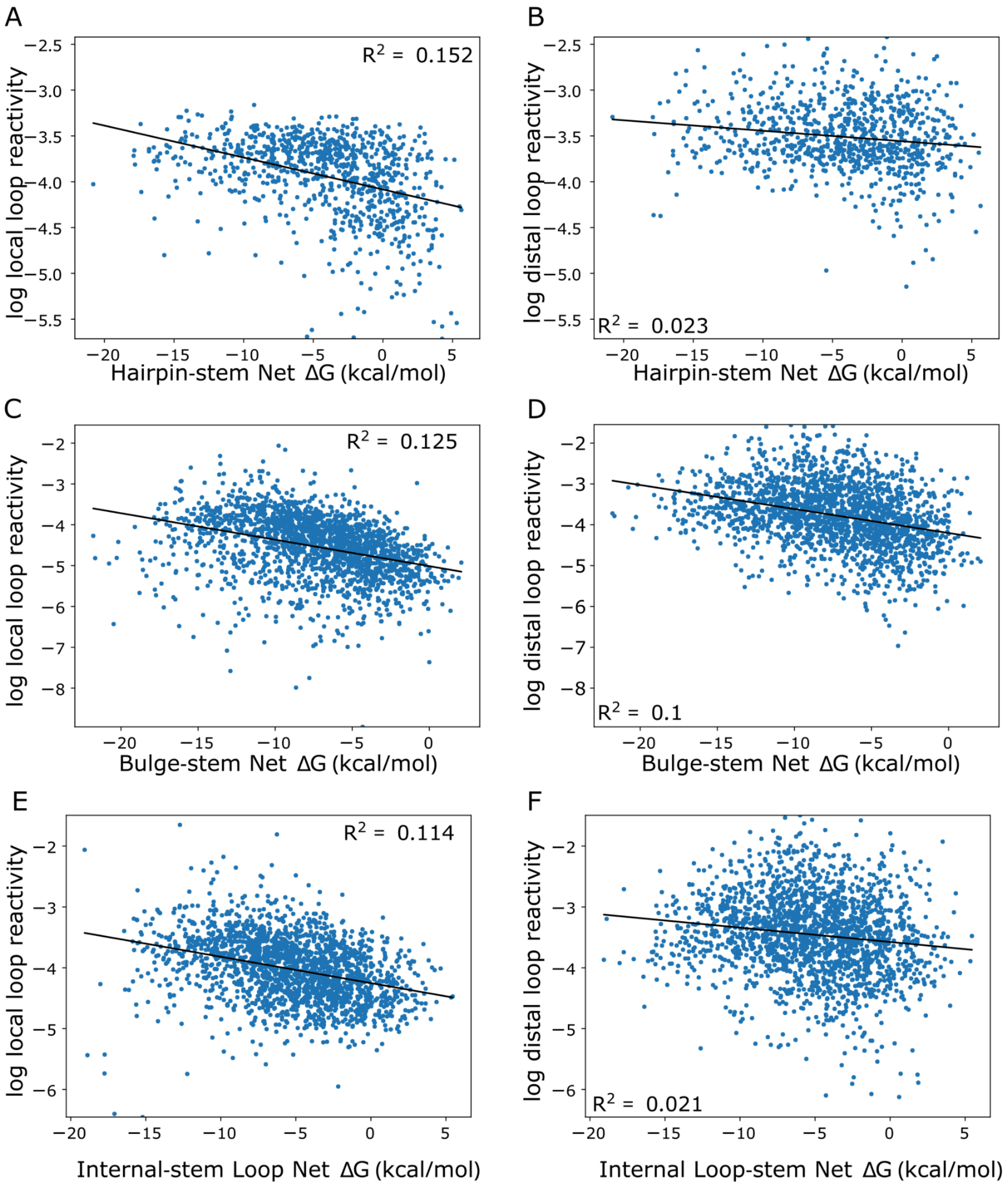

Supplementary Figure 5. Loop reactivities are invariant to loop net ΔGs. Hairpin-stem net ΔG versus local (A) and distal (B) average loop reactivities. Bulge-stem net ΔG versus local (C) and distal (D) average loop reactivities. Internal loop-stem net ΔG versus local (E) and distal (F) average loop reactivities.

	K	n	A
Hairpin loops	18.6	4.39	1.
Bulges	17.9	2.71	1.
Internal loops	21.2	2.85	1.

Supplementary Table 1. Fit parameters for curve fitting to the Hill Equation.
